# Supplementary figures and images for: Sarcopenia as an important determinant for adverse outcomes in patients with pyogenic liver abscess
Source: PeerJ. 2023 Oct 4;11:e16055. doi: 10.7717/peerj.16055 (PMC10559880; doi:10.7717/peerj.16055)

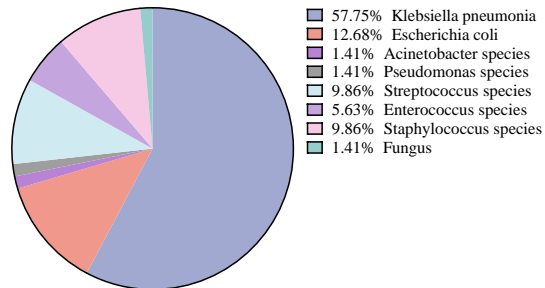

**Total(n=71)**

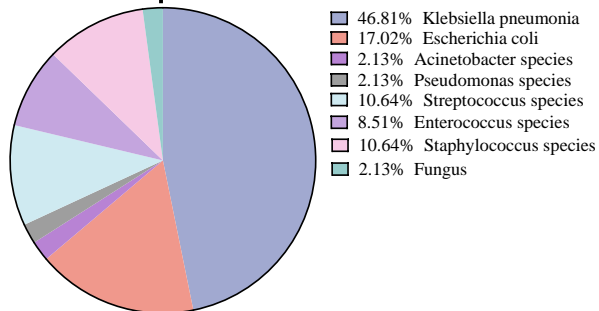

**Sarcopenia group(n=47)**

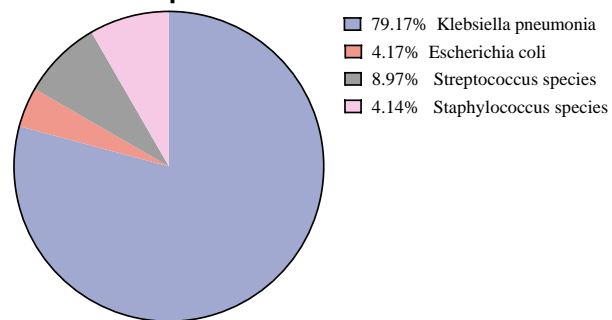

**Non-sarcopenia group(n=24)**

Supplement: Supplemental Information 1 [file peerj-11-16055-s001.pdf]
