# Supplementary material for: Sarcopenia as an important determinant for adverse outcomes in patients with pyogenic liver abscess
Source: PeerJ. 2023 Oct 4;11:e16055. doi: 10.7717/peerj.16055 (PMC10559880; doi:10.7717/peerj.16055)
Supplement: Supplemental Information 2 [file peerj-11-16055-s002.docx]

| **Table S1** Univariate logistic regression analysis for risk factors associated with adverse outcomes (serious complications or mortality) in patients with pyogenic liver abscess | | | | | | |
| --- | --- | --- | --- | --- | --- | --- |
|  | **Total** | *P* | **Male** | *P* | **Female** | *P* |
| **Variables** | OR (95% CI) |  | OR (95% CI) |  | OR (95% CI) |  |
| Age | 1.029 (1.003,1.055) | **0.030** | 1.038 (1.006, 1.071) | **0.019** | 1.016 (0.967, 1.067) | 0.526 |
| Male/Female | 1.719 (0.834, 3.542) | 0.142 | - | - | - | - |
| Hemoglobin, g/L | 1.005 (0.990, 1.020) | 0.545 | 1.007 (0.988, 1.026) | 0.490 | 0.982 (0.949, 1.016) | 0.287 |
| Lymphocyte count, 10^9^/L | 1.002 (0.771, 1.302) | 0.989 | 1.090 (0.788, 1.507) | 0.064 | 0.392 (0.039, 1.107) | 0.077 |
| BMI | 0.933 (0.848, 1.026) | 0.153 | 0.926 (0.829, 1.035) | 0.176 | 0.967 (0.784, 1.192) | 0.751 |
| Random blood glucose, mmol/L | 1.009 (0.932, 1.094) | 0.818 | 1.059 (0.950, 1.180) | 0.304 | 0.900 (0.764, 1.059) | 0.205 |
| cTnI , pg/mL | 0.995 (0.987, 1.004) | 0.319 | 0.995 (0.986, 1.004) | 0.288 | 1.369 (0.816, 2.297) | 0.235 |
| Platelet count, 10^9^/L | 0.998 (0.995, 1.000) | 0.074 | 1.000 (0.997, 1.002) | 0.729 | 0.991 (0.985, 0.998) | **0.009** |
| ALT, U/L | 1.092 (1.056, 1.129) | **<0.001** | 1.094 (1.050, 1.139) | **<0.001** | 1.086 (1.023, 1.153) | **0.007** |
| AST, U/L | 1.124 (1.073, 1.178) | **<0.001** | 1.128 (1.067, 1.194) | **<0.001** | 1.116 (1.020, 1.220) | **0.016** |
| PT, s | 1.399 (1.085, 1.803) | **0.010** | 1.168 (0.857, 1.591) | 0.325 | 1.953 (1.172, 3.255) | **0.010** |
| Albumin, g/L | 0.901(0.847, 0.960) | **0.001** | 0.898 (0.828, 0.975) | **0.010** | 0.894 (0.805, 0.992) | **0.035** |
| Total bilirubin, umol/L | 1.099 (1.036, 1.166) | **0.002** | 1.090 (1.022, 1.163) | **0.009** | 1.113 (0.970, 1.277) | 0.128 |
| CRP, mg/L | 1.010 (1.004, 1.017) | **0.001** | 1.012 (1.004, 1.02) | **0.003** | 1.007 (0.997, 1.018) | 1.183 |
| PCT, ng/ml | 1.089 (1.007, 1.176) | **0.032** | 1.100 (1.002, 1.206) | **0.044** | 0.999 (0.803, 1.241) | 0.990 |
| Creatinine, umol/L | 1.042 (1.021, 1.063) | **<0.001** | 1.047 (1.018, 1.076) | **0.001** | 1.003 (0.996, 1.071) | 0.082 |
| BUN, mmol/L | 1.362 (1.122, 1.652) | **0.002** | 1.281 (1.025, 1.599) | **0.029** | 1.488 (0.983, 2.253) | 0.060 |
| TC, mmol/L | 0.549 (0.376, 0.800) | **0.002** | 0.710 (0.454, 1.110) | 0.133 | 0.283 (0.122, 0.655) | **0.003** |
| TG, mmol/L | 1.281 (0.505, 3.278) | 0.605 | 3.656 (0.596, 22.442) | 0.161 | 0.475 (0.098, 2.299) | 0.355 |
| Pleural effusion | 3.137 (1.572, 6.256) | **0.001** | 2.782 (1.208, 6.410) | **0.016** | 5.143 (1.384, 19.107) | **0.014** |
| Diabetes | 0.952 (0.390, 2.327) | 0.915 | 1.906 (0.583, 6.231) | 0.286 | 0.122 (0.013, 1.152) | 0.066 |
| Cardiovascular disease | 2.789 (0.317, 24.509) | 0.355 | 1.397 (0.140, 13.929) | 0.776 | - | 0.999 |
| Hypertension | 1.785 (0.705, 4.517) | 0.221 | 1.599 (0.482, 5.311) | 0.443 | 2.667 (0.597, 11.915) | 0.199 |
| Liver and gallbladder stones | 1.103 (0.489, 2.488) | 0.814 | 2.000 (0.679, 5.888) | 0.208 | 0.310 (0.066, 1.457) | 0.138 |
| The size of abscess (≥5cm) | 1.115 (0.524, 2.374) | 0.778 | 1.077 (0.432, 2.684) | 0.874 | 1.560 (0.368, 6.618) | 0.546 |
| Gas formation | 1.464 (0.493, 4.352) | 0.492 | 0.912 (0.255, 3.257) | 0.887 | 4.737 (0.503, 44.572) | 0.174 |
| *Klebsiella pneumonia** | 1.120 (0.376, 3.340) | 0.839 | 1.923 (0.554, 6.670) | 0.303 | 0.143 (0.010, 1.995) | 0.148 |
| SMA | 0.998 (0.987, 1.010) | 0.749 | 0.987 (0.970, 1.003) | 0.107 | 0.998 (0.962, 1.035) | 0.911 |
| SMI | 0.941 (0.900, 0.983) | **0.006** | 0.913 (0.859, 0.971) | **0.004** | 0.883 (0.782, 0.996) | **0.021** |
| SMD | 0.949 (0.909, 0.991) | **0.018** | 0.923 (0.870, 0.979) | **0.008** | 0.951 (0.885, 1.023) | 0.178 |
| SMG | 0.999 (0.998, 1.000) | **0.001** | 0.998 (0.998, 0.999) | **<0.001** | 0.998 (0.996. 1.000) | **0.015** |
| IMAT | 1.052 (0.985, 1.124) | 0.131 | 1.097 (1.002, 1.202) | **0.046** | 0.989 (0.883, 1.107) | 0.842 |
| VFA | 1.002 (0.998, 1.007) | 0.273 | 1.002 (0.997, 1.006) | 0.535 | 1.004 (0.991, 1.016) | 0.562 |
| SFA | 1.000 (0.995, 1.005) | 0.959 | 1.002 (0.994, 1.010) | 0.602 | 1.001 (0.993, 1.010) | 0.724 |
| MFI | 1.452 (0.794, 2.656) | 0.226 | 1.395 (0.668, 2.911) | 0.375 | 0.463 (0.060, 3.603) | 0.462 |
| VAI | 1.004 (0.991, 1.016) | 0.555 | 1.003 (0.989, 1.017) | 0.689 | 1.000 (0.969, 1.033) | 0.982 |
| SAI | 0.995 (0.981, 1.009) | 0.452 | 1.003 (0.980, 1.026) | 0.813 | 0.997 (0.976, 1.018) | 0.767 |
| Abdominal wall fat thickness | 0.979 (0.936, 1.025) | 0.372 | 1.021 (0.951, 1.096) | 0.560 | 0.963 (0.894, 1.037) | 0.316 |
| Intra-abdominal fat thickness | 1.006 (0.993, 1.019) | 0.361 | 1.004 (0.989, 1.020) | 0.613 | 1.000 (0.968, 1.032) | 0.994 |
| Antibiotics plus percutaneous | 1.973 (1.000, 3.892) | 0.050 | 1.728 (0.754, 3.961) | 0.196 | 2.292 (0.670, 7.839) | 0.186 |
| **Note:***There were a total of 71 patients with positive microbial culture.  **Abbreviations:** OR, odd ratio ;CI: confidence interval; ALT, alamine aminotransferase; AST, aspartate aminotransferase; PT, prothrombin time; CRP, C-reactive protein; PCT, procalcitonin; BUN, blood urea nitrogen; TC, total cholesterol；TG, Triglyceride;;SMA, skeletal muscle area; SMI, skeletal muscle index; SMD, skeletal muscle density; SMG, SMG is defined as the product of SMI and SMD. IMAT, intramuscular adipose tissue; VFA, visceral fat area; SFA, subcutaneous fat area; MFI, mesenteric fat index; VAI; visceral adipose index; SAI, subcutaneous adipose index | | | | | | |
