# Supplementary material for: Sarcopenia as an important determinant for adverse outcomes in patients with pyogenic liver abscess
Source: PeerJ. 2023 Oct 4;11:e16055. doi: 10.7717/peerj.16055 (PMC10559880; doi:10.7717/peerj.16055)
Supplement: Supplemental Information 3 [file peerj-11-16055-s003.docx]

| **Table S2** Multivariate logistic regression analysis for risk factors associated with adverse outcomes (serious complications or mortality) in a subgroup of patients with microbial culture results. | | |
| --- | --- | --- |
| **Variables** | **OR (95% CI)** | **P** |
| Age | 1.018 (0.963,1.076) | 0.527 |
| Male/Female | 6.127 (0.615-61.039) | 0.122 |
| Total bilirubin | 1.024 (0.967-1.085) | 0.413 |
| Albumin | 0.927 (0.807-1.064) | 0.281 |
| Creatinine | 1.043 (0.992-1.096) | 0.101 |
| Pleural effusion | 1.940 (0.481-7.819) | 0.352 |
| *Klebsiella pneumoniae* | 1.719 (0.378-7.881) | 0.483 |
| SMI | 0.839 (0.739-0.951) | **0.006** |
| **Notes**: The sample size of this subgroup was 71. The covariates included in the multivariable logistic regression analysis were age, gender, total bilirubin, albumin, creatinine, pleural effusion, *Klebsiella pneumoniae*, SMI.  **Abbreviations**: SMI, skeletal muscle index. | | |
